# Bioactive natural compounds as potential medications for osteogenic effects in a molecular docking approach

**DOI:** 10.3389/fphar.2022.955983

**Published:** 2022-08-24

**Authors:** Yuqiong Wu, Yulan Liu, Yuanjin Xu, Ao Zheng, Jiahui Du, Lingyan Cao, Junfeng Shi, Xinquan Jiang

**Affiliations:** ^1^ Department of Prosthodontics, Shanghai Ninth People’ s Hospital, Shanghai Jiao Tong University School of Medicine, College of Stomatology, Shanghai Jiao Tong University, Shanghai, China; ^2^ National Center for Stomatology, National Clinical Research Center for Oral Diseases, Shanghai Key Laboratory of Stomatology, Shanghai Engineering Research Center of Advanced Dental Technology and Materials, Shanghai, China; ^3^ Department of Oral Surgery, Shanghai Ninth People’s Hospital, Shanghai Jiao Tong University School of Medicine, College of Stomatology, Shanghai Jiao Tong University, Shanghai, China

**Keywords:** icariin, bioinformatic analysis, hydroxycholesterol, ginsenoside Rb1, bone repair

## Abstract

Bone defect repair and fracture healing are critical challenges in clinical treatments. Bioactive natural compounds are potential resources for medications for osteogenic effects. We have identified icariin, the effective ingredient of *Epimedium pubescens*, to promote osteogenic differentiation of bone mesenchymal stem cells (BMSCs) and repair bone defects. To explore more natural compounds with the potential modality for bone repair, in the present study, we employed an icariin-induced gene expression pattern as an osteogenic model and screened the Connectivity Map database for small molecules with gene expression signatures similar to this model. We verified the effectiveness of this molecule docking approach by introducing hydroxycholesterol, the second highest score of the similarity to icariin, into the osteoinductive experiments *in vitro* and demonstrated its excellent osteogenic effect on BMSCs compared with a BMP-2-positive control group. Based on the compatible result of hydroxycholesterol, subsequently, ginsenoside Rb1 was chosen as the most drug-like natural compound among the molecule docking results from icariin. Finally, ginsenoside Rb1 was demonstrated to promote the expression of osteoblastic genes and ALP activity *in vitro* and repair the calvarial defect of rats *in vivo*. The study aimed to provide diverse choices for clinical application in bone repair and functional regeneration.

## Introduction

Bone defects caused by trauma, tumors, degenerative diseases, etc., have a severe impact on daily life, especially in the aging population (Salhotra et al., 2020). Nowadays, tissue engineering materials with medications or growth factors provide a good choice for the regeneration of bone defects (Yin et al., 2019) since a range of osteo-inductive growth factors has already been shown to be effective to promote bone repair in animal studies. However, due to the high price and high-quality preservation conditions, as well as the limited active periods, the clinical application of growth factors has been limited. Therefore, there is an urgent need to develop suitable surrogate molecules with a better safety profile and lower cost than growth factors.

Natural compounds are a considerable strategy for meeting the demands for healing bone fractures and defects (Bose and Sarkar, 2020). Due to their natural abundance and good cost-effectiveness, traditional Chinese medicine compounds have great potential in the field of bone regeneration. Over recent years, the active ingredients extracted from medicinal herbs involved in traditional Chinese medicine have been studied for the potential application in osteogenic effects. The compounds include naringin (Zhao et al., 2022), curcumin (Sarkar and Bose, 2019), quercetin (Ren et al., 2022), berberine (Jia et al., 2019), resveratrol (Min et al., 2020), and salvianolic acids (Ji et al., 2019), which boost bone regeneration by promoting bone formation or inhibiting bone resorption.

Recently, we have identified that icariin (C33H40O15; molecular weight: 676.67), the major active ingredient of Herba Epimedii, was the standard for its quality control (Cheung et al., 2009). It could correct the decrease of estrogen in the serum and partly restore the decreased weight of the uterus in ovariectomized (OVX) rats (Nian et al., 2009). Furthermore, we have identified that icariin could repair bone defects in rats with osteoporosis (Wu et al., 2017).

To investigate more natural compounds as a potential modality for bone repair, in the present study, we employed the gene expression pattern, followed by the administration of icariin as an osteogenic model, and by comprehensive prediction integrated with bioinformatic analysis, we screened a number of compounds and verified that one of the representative compounds, hydroxycholesterol, acquiring the second highest score of the gene expression similarity to icariin, has a great osteoinductive effect on BMSCs using BMP-2 as a positive control group. Furthermore, we chose another representative, ginsenoside Rb1, which shows a gene expression pattern similar to icariin as well, and found it has excellent osteogenic effects *in vitro* and *in vivo*. The study aimed to provide a novel natural small molecular screening method for osteogenic drug discovery and supply more diverse choices for clinical application in bone repair and functional regeneration.

## Methods and materials

### Cell culture

For the analysis, 4-week-old male Sprague–Dawley rats (Shanghai SLAC Experimental Animal Center, Shanghai, China), weighing about 75 g, were used for bone mesenchymal stem cell (BMSC) isolation and culture. Briefly, the bone marrows of femurs and tibia were flushed out with Dulbecco’s modified Eagle’s medium (DMEM; HyClone, South Logan, UT, United States) supplemented with 100 unit/ml penicillin and 100 μg/ml streptomycin (HyClone). After centrifugation at 1500 rpm for 5 min, the precipitate was then mixed with complete DMEM supplemented with 10% fetal bovine serum (FBS; HyClone) and cultured at 37°C in a humidified 5% CO_2_ incubator. Non-adherent cells were removed by changing the fresh medium every 3 days. When large colonies formed and became confluent, the primary rat BMSCs were passaged. The BMSCs from passages 2–3 were used for the experiments.

### Extraction of total RNA and microarray processing and RNA sequencing

BMSCs were plated on 6-well plates at 2 × 105 cells/well and incubated for 24 h, followed by incubation with icariin or ginsenoside Rb1 at the final concentrations of 20 μM, respectively. Total RNA was isolated from the cells after 24 h of icariin treatment using the TRIzol reagent (Invitrogen, Carlsbad, United States), according to the manufacturer’s recommended protocol. RNA quantity and quality were measured by using the NanoDrop ND-1000 spectrophotometer. RNA integrity was assessed by standard denaturing agarose gel electrophoresis. Rat 12 × 135K gene expression array was manufactured by Roche NimbleGen. ds-cDNA was cleaned and labeled, in accordance with the NimbleGen Gene Expression Analysis protocol (NimbleGen Systems, Inc., Madison, WI, United States). Briefly, ds-cDNA was incubated with 4 μg RNase A at 37°C for 10 min and cleaned using phenol:chloroform:isoamyl alcohol, followed by ice-cold absolute ethanol precipitation. The purified cDNA was quantified using a NanoDrop ND-1000 spectrophotometer. For Cy3 labeling of cDNA, the NimbleGen One-Color DNA labeling kit was used according to the manufacturer’s guidelines detailed in the gene expression analysis protocol (NimbleGen Systems, Inc., Madison, WI, United States). Expression data were normalized through quantile normalization and the Robust Multichip Average (RMA) algorithm included in NimbleScan software. The probe level files and gene level files were generated after normalization. All gene level files were imported into Agilent GeneSpring GX software (version 11.5.1). Differentially expressed genes were identified through fold change filtering. Hierarchical clustering was performed by Agilent GeneSpring GX software (version 11.5.1). Gene ontology (GO) analysis and Kyoto Encyclopedia of Genes and Genomes (KEGG) pathway analysis were performed using the standard enrichment computation method with GoStats/clusterProfiler packages. *p* < 0.05 was considered statistically significant. According to ginsenoside Rb1, BMSCs were plated on 6-well plates at 2 × 105 cells/well and incubated for 24 h, followed by incubation with ginsenoside Rb1 at a final concentration of 20 μM. RNA-Seq high-throughput sequencing and subsequent bioinformatics analysis were all performed by Cloud-Seq Biotech (Shanghai, China). Total RNA (1 μg) was used for removing the rRNAs using Ribo-Zero rRNA removal kits (Illumina, San Diego, CA, United States), following the manufacturer’s instructions. RNA libraries were constructed using rRNA-depleted RNAs with the TruSeq Stranded Total RNA Library Prep kit (Illumina, San Diego, CA, United States), according to the manufacturer’s instructions. Libraries were controlled for quality and quantified using the BioAnalyzer 2100 system (Agilent Technologies, Inc., United States ). Ten pM libraries were denatured as single-stranded DNA molecules, captured on Illumina flow cells, amplified *in situ* as clusters, and finally sequenced for 150 cycles on the Illumina HiSeq sequencer, according to the manufacturer’s instructions.

### Pharmacological network analysis

Genes related to osteogenesis and angiogenesis were selected from the GeneCards database (https://www.genecards.org/). The ingredients and their targeted genes were downloaded from the Traditional Chinese Medicine Systems Pharmacology (TCMSP) database (https://tcmspw.com/tcmsp.php). The network analysis of protein–protein interaction (PPI) was performed in the STRING database, version 11.0b (https://string-db.org/).

### Evaluating the effect of hydroxycholesterol on cell viability

Briefly, BMSCs were treated with DMEM (HyClone, United States ) containing 1, 2, 5, 10, 20, and 40 μM hydroxycholesterol (MedChemExpress, New Jersey, United States ); normal DMEM was used as a blank control. The cells were seeded at a density of 1 × 104 cells per well in the 96-well plates. After 6 h, the culture medium was replaced with the aforementioned hydroxycholesterol-containing medium. After incubation for 24 h, cell viability was detected with cell counting kit-8 (Dojindo, Japan), according to the manufacturer’s instructions, and the optical density (OD) was measured at 450 nm (OD450) to calculate the cell relative viability. Cell relative viability = (ODexperiment-ODblank)/(ODcontrol-ODblank) × 100%.

### Alkaline phosphatase staining and activity

BMSCs at 5 × 10^4^ cells/well were cultured in 24-well plates. When the cell reached 90% confluence, the medium was replaced with DMEM containing hydroxycholesterol at various concentrations (0, 1, 2, and 5 μM). Meanwhile, the osteogenic growth factor BMP-2 (100 ng/ml, PeproTech, New Jersey, United States ) was used as a positive control (po in short). Alkaline phosphatase (ALP) staining and activity quantitation were performed on days 3 and 7 after treating with hydroxycholesterol and BMP-2, respectively. Furthermore, BMSCs at 5 × 10^4^ cells/well were cultured overnight in 24-well plates. ALP staining and activity quantitation were performed on day 4 after treating with ginsenoside Rb1 at concentrations of 0, 10, 20, and 40 μM, respectively. For ALP staining, each sample was fixed in 4% paraformaldehyde (PFA) for 10 min and then incubated with a substrate solution from an ALP staining kit (Beyotime), according to the manufacturer’s protocol. After staining, the results were observed using a digital camera (ECLIPSE TS100, Nikon, Tokyo, Japan). The ALP activity was assayed using the Alkaline Phosphatase Assay Kit (Beyotime, Suzhou, China), following the manufacturer’s protocol. The absorbance values (OD) at 405 nm were measured to determine the ALP activity. Total protein contents were assessed using a PierceTM BCA Protein Assay Kit (Thermo Fisher Scientific, Waltham, MA, United States ). OD values were normalized to the bovine serum albumin standard curve at 562 nm. The ALP activity was accessed as an OD value at 405 nm per milligram of total protein.

### Immunofluorescence staining

The BMSCs were implanted in a confocal dish at a density of 10^5^/ml. Then, 24 h later, the medium was replaced with normal DMEM, hydroxycholesterol-containing DMEM, and BMP-2-containing DMEM, respectively. Immunofluorescence staining assay was carried out on day 10. Briefly, samples were fixed with 4% PFA and rinsed in PBS after infiltrating and blocking; specific primary mouse monoclonal antibodies against osteocalcin (OCN, Santa Cruz Biotechnology, United States ) were used overnight at 4ºC, followed by Alexa Fluor 594 Donkey Anti-Mouse IgG (H + L) secondary antibodies (YEASEN, China) for 1 h at room temperature. Fluorescein isothiocyanate (FITC)-phalloidin (YEASEN, China) was applied to show the cell skeleton, while the nuclei were stained with DAPI (Sigma, United States). Confocal laser scanning microscopy (CLSM, Leica, Germany) was used for subsequent observation. The fluorescence intensity was analyzed by ImageJ.

### Identifying icariin-like ingredients using connectivity map (CMAP)

CMAP is a database with a collection of gene expression data sets from cultured human cell lines treated with various small molecules (https://clue.io/). Users can search the CMAP database with two lists of genes (referred to as ‘signatures’ and obtained from any experimental conditions): one in which genes are upregulated and the other in which genes are downregulated (seen in Supplementary Material S1). CMAP reports enrichment scores (which lie between −100 and 100) of all the drugs on the basis of relative correlations between query signatures and reference gene expression profiles of individual drugs in the CMAP database (seen in Supplementary Material S2). Higher positive scores for a given signature indicate that the experimental results of the particular drug treatment in CMAP show a gene expression profile similar to the signature that is provided by the user.

### Osteoblastic gene expression by qRT-PCR

BMSCs at 1 × 10^5^ cells/well were cultured in 12-well plates. When the cell reached 90% confluence, the medium was replaced with DMEM-containing hydroxycholesterol (2 μM) and BMP-2 (100 ng/ml, PeproTech, New Jersey, United States), respectively. The osteogenic growth factor BMP-2 was used as a positive control. After incubating for 3 and 7 days, total RNA from each sample was extracted with RNAiso Plus (TaKaRa, Japan), following the manufacturer’s protocol.

Furthermore, BMSCs plated on 6-well plates were incubated with ginsenoside Rb1 at final concentrations of 0, 10, 20, and 40 μM, respectively. Total RNA was isolated from the cells after 3 and 7 days of treatment with the TRIzol reagent (Invitrogen, United States), according to the manufacturer’s recommended protocol.

Complimentary DNA (cDNA) was synthesized by means of a cDNA Synthesis Reverse Transcription Kit (Fermentas, Thermo, United States). Real-time PCR assay for runt-related transcription factor 2 (Runx2), alkaline phosphatase (ALP), collagen I (Col-1), osteopontin (OPN), and osteocalcin (OCN) were performed using a Light-Cycler system with SYBR Premix Ex TaqTM (TaKaRa, Japan), according to the manufacturer’s instructions (Wu et al., 2015a). Each sample was analyzed in triplicate. The primer sequences (Sangon Biotech, China) used in the present study are listed in Table 1.

**TABLE 1:** List of primers used in the RT-PCR.

Gene	Primer sequence (5′→3′)	Length
Gapdh	Forward: GAC​ATC​AAG​AAG​GTG​GTG​AAG​C	22
	Reverse: TGT​CAT​TGA​GAG​CAA​TGC​CAG​C	22
Runx2	Forward: CAC​AAG​TGC​GGT​GCA​AAC​TT	20
	Reverse: CTT​GCA​GCC​TTA​AAT​GAC​TCG​G	22
ALP	Forward: CTC​CTT​AGG​GCC​ACC​GCT​C	19
	Reverse: GAG​ATC​CGT​TCC​TCG​CTG​GA	20
Col-1	Forward: CAC​TGC​AAG​AAC​AGC​GTA​GC	20
	Reverse: ACA​AGC​GTG​CTG​TAG​GTG​AA	20
OPN	Forward: GCT​GAA​TTC​TGA​GGG​ACC​AAC​T	22
	Reverse: CAA​ACT​CAG​CCA​CTT​TCA​CCG	21
OCN	Forward: GAA​TAG​ACT​CCG​GCG​CTA​CC	20
	Reverse: TCC​TGG​AAG​CCA​ATG​TGG​TC	21

### Animal surgery

A 5-mm diameter full-thickness rat cranial bone defect is a common model for evaluating the bone-forming capacity of the engineered complexes *in vivo*. Twelve female Sprague–Dawley rats aged 12 weeks were obtained from the Ninth People’s Hospital Animal Center (Shanghai, China) for a cranial defect repair experiment, which was approved by the Animal Experimental Ethical Inspection Shanghai Ninth People’s Hospital affiliated to the Shanghai Jiao Tong University, School of Medicine (HKDL[2016]321). The animals were anesthetized by an intraperitoneal injection of pentobarbital (Nembutal 3.5 mg/100 g). A 1.5-cm sagittal incision was made on the scalp, and the calvarium was exposed by blunt dissection. Two critical-sized defects were created by means of a 5-mm diameter trephine bur (Fine Science Tools, Foster City, CA, United States ). Thereafter, the rats were divided into four groups, including silk fibroin hydrogel gel containing no ginsenoside Rb1-loaded HAp granules (group A, silk/HAp, *n* = 3), silk fibroin hydrogel gel containing ginsenoside Rb1-loaded HAp granules (group B, silk/HAp/Rb1, *n* = 3), BMSC-loaded silk fibroin hydrogel gel containing no ginsenoside Rb1-loaded HAp granules (group C, BMSCs/silk/HAp, *n* = 3), and BMSC-loaded silk fibroin hydrogel gel containing ginsenoside Rb1-loaded HAp granules (group D, BMSCs/silk/HAp/Rb1, *n* = 3). The composite silk fibroin hydrogel gel with ginsenoside Rb1-loaded HAp was prepared as described in our previous study (Wu et al., 2022).

### Micro-computerized tomography measurements

The rats in each group were all sacrificed 8 weeks after cranial surgery, and the specimens were fixed in formaldehyde solution. Then, the samples of each group (*n* = 3 for each group) were examined using a micro-CT system (mCT-80, Scanco Medical AG, Switzerland), as described in our previous study (Wu et al., 2015b). The amount of the newly formed bone was calculated by dividing the newly formed bone voxels by the total voxels of the initially implanted micro/nano HAp granule volume. Moreover, three-dimensional images were reconstructed, and the percentage object surface and the trabecular number in the bone defect were calculated by using an auxiliary histomorphometric software application (SCANCO Medical AG, Switzerland).

### Histological and histomorphometric observations

The cranial samples were dehydrated in an ascending concentration of alcohol from 70% to 100% and then embedded in polymethylmethacrylate (PMMA). Three longitudinal sections for each specimen were prepared, as described in our previous study (Wu et al., 2015b). First, the samples were observed for fluorescent labeling using CLSM (Leica TCS, Germany), and fluorochrome staining for new bone formation and mineralization was quantified. The data on yellow (TE), red (AL), and green (CA) represent the bone regeneration and mineralization at weeks 2, 4, and 6 after operation, respectively. Finally, the samples were stained with van Gieson’s picro fuchsin for histological observation. The area of newly formed bone was quantified from the serial section collected from each sample, using a personal computer-based image analysis system (Image-Pro Plus 6.0, Media Cybernetic, United States), and reported as a percentage of the whole bone defect area, respectively.

### Statistical analysis

In this study, the results of repeated experiments were shown as the mean ± standard deviation (SD). Using SPSS 17.0 (SPSS Science) software, the significant differences between data sets were analyzed using one-way analysis of variance (ANOVA), respectively, and indicated by *p < 0.05.

## Results

### Osteogenic bioactive ingredient screening and identification

**FIGURE 1:**
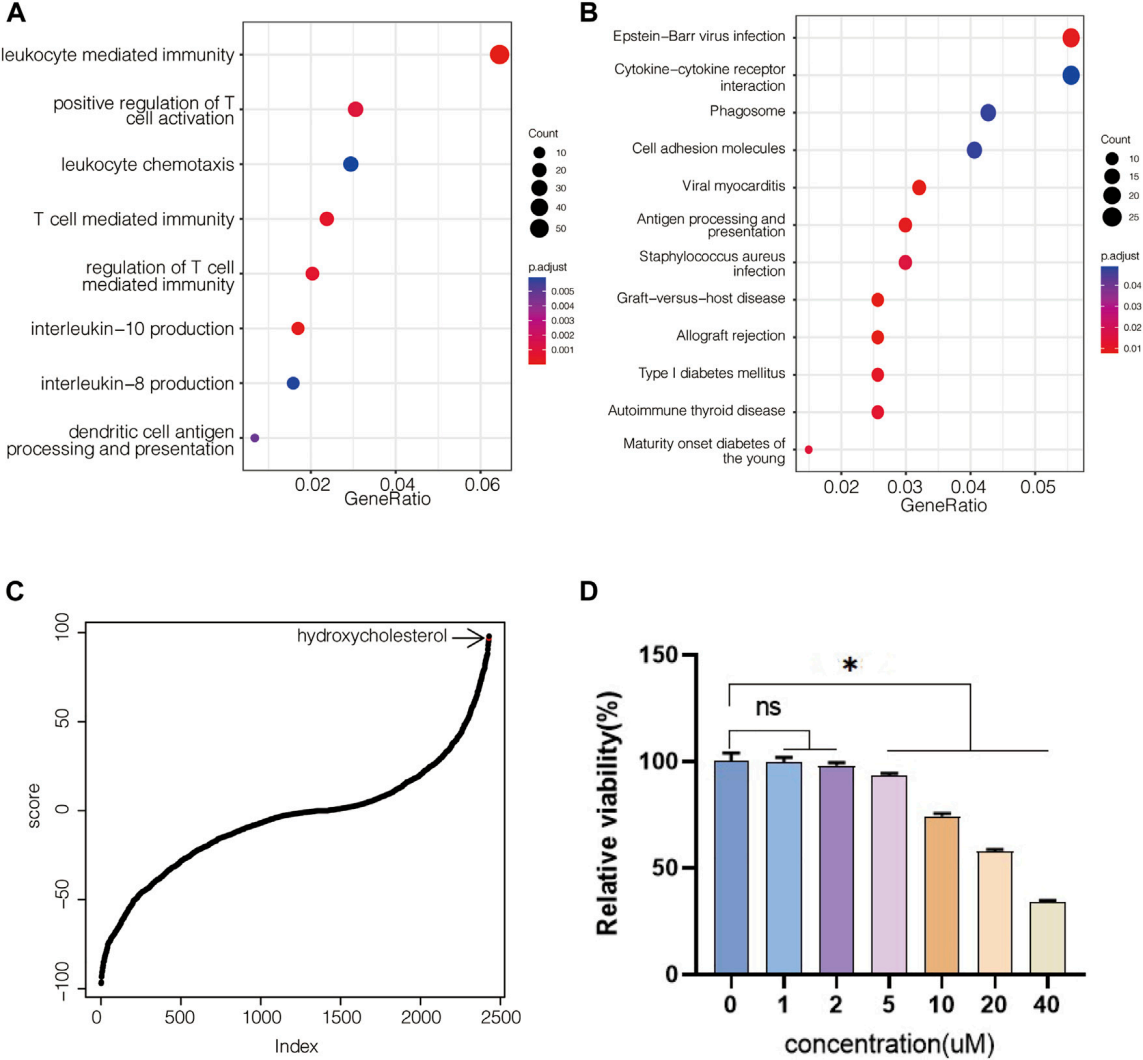
Molecular screening based on the icariin-induced gene expression signature. **(A)** GO-based gene set enrichment analysis of BMSCs administered with icariin at 20 μM after 24 h; **(B)** KEGG-based gene set enrichment analysis of BMSCs administered with icariin; **(C)** hydroxycholesterol is involved in CMap-based molecular screening results from the icariin-induced gene expression signature; **(D)** cell viability of BMSCs administered with different concentrations of hydroxycholesterol (ns, not significant; *, *p* < 0.05).

Icariin has been identified as an osteogenic compound in our previous study (Wu et al., 2017), (Wu et al., 2015a). To investigate the molecular mechanisms of the osteogenic effects of icariin, we performed gene expression profile analysis on BMSCs administered by icariin at 20 μM. By extracting the differentially expressed genes, it was identified that the administration of icariin resulted in an immunoregulation-type gene expression profile (Figures 1A,B). Since icariin has been identified as an osteogenic potential in the previous study (Wu et al., 2017; Wu et al., 2015a), the icariin-induced gene expression signature was defined as an osteogenic transcription model (Supplementary Material S1). Based on this model, we screened the CMap database for small molecules with gene expression signatures similar to this model (Supplementary Material S2). To confirm that this is an applicable approach for finding osteogenic compounds similar to icariin, hydroxycholesterol, which has the osteogenic potential and the second highest score of similarity to icariin, was chosen to be verified (Figure 1C). First, for evaluating the effect of hydroxycholesterol on cell viability, BMSCs were cultured with a medium containing varying concentrations of hydroxycholesterol to determine the appropriate concentration for cell survival. The cell viability was unaffected at hydroxycholesterol concentrations less than 5 μM but decreased significantly when the concentration exceeded 10 μM (Figure 1D). Therefore, hydroxycholesterol concentrations under 5 μM were selected for subsequent experiments.

### Hydroxycholesterol induced BMSC osteogenic differentiation

**FIGURE 2:**
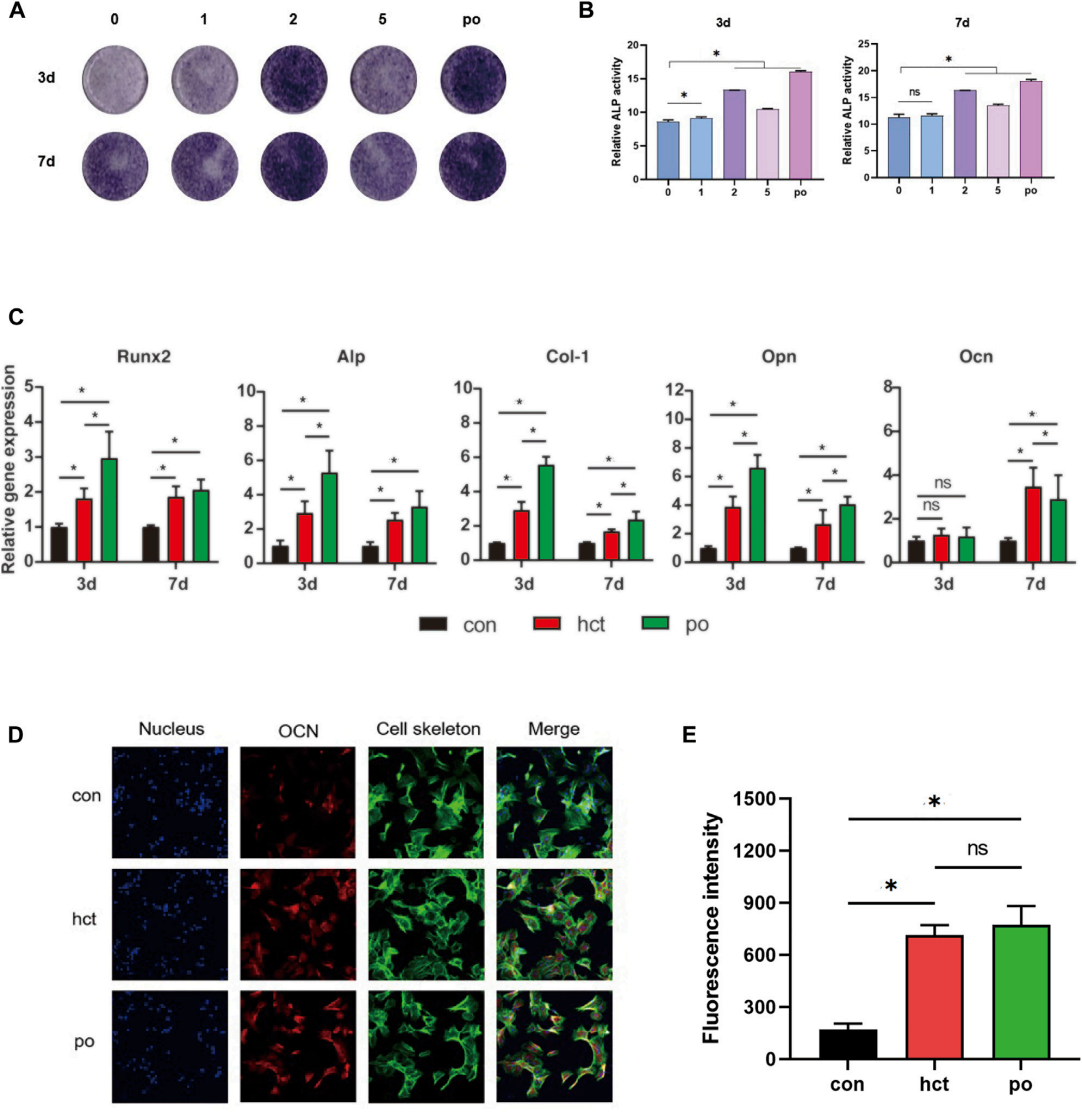
Osteogenic effect of hydroxycholesterol. **(A,B)** ALP staining **(A)** and ALP quantitative activity assay **(B)** of BMSCs administered with different concentrations of hydroxycholesterol and BMP-2 as the positive group (po in short) at days 3 and 7. **(C)** Expression of osteogenesis-related genes in BMSCs, followed by administration of hydroxycholesterol (2 μM) and BMP-2 at days 3 and 7; **(D,E)** immunostaining **(D)** and fluorescence intensity **(E)** of OCN in BMSCs, followed by administration of hydroxycholesterol (2 μM) and BMP-2 at day 7 (ns, not significant; *, *p* < 0.05).

To investigate the osteogenic effect of hydroxycholesterol, the ALP activity of the BMSCs treated by hydroxycholesterol in a series of concentrations was investigated at day 3 and day 7. It showed that the ALP activity was upregulated by various concentrations of hydroxycholesterol compared with the medium control, indicating that hydroxycholesterol could promote osteogenic differentiation of BMSCs (Figures 2A,B). It is worth mentioning that the ALP activity significantly increased in the 2-μM group. Moreover, a similar tendency was observed in the ALP semiquantitative analysis. Therefore, it is speculated that 2 μM is the optimal concentration of hydroxycholesterol to promote the osteogenic differentiation of BMSCs.

To further evaluate the osteogenic inductive effects of 2 μM hydroxycholesterol, several pivotal osteogenesis-related gene expressions were detected by RT-PCR. With the stimulation, the expressions of Runx2, ALP, Col-1, and OPN increased significantly in 3 days and 7 days (Figure 2C). At day 7, the expression level of the key osteogenic marker OCN in BMSCs also increased significantly in the experimental group, which was slightly higher than that of the positive control group, although the difference between the two was not statistically significant (Figure 2C). The OCN protein level detected by immunofluorescence staining assay after cell treatment was also used to measure the effectiveness of osteogenic induction. Compared with the DMEM control group, the OCN protein level of the hydroxycholesterol group increased significantly, with a similar trend to the BMP-2-positive control group (Figures 2D,E). All these findings demonstrated that hydroxycholesterol had an excellent osteogenic effect on BMSCs, and 2 μM is optimal for constructing an osteogenic microenvironment.

### Ginsenoside Rb1 as a potential osteogenic bioactive ingredient

**FIGURE 3:**
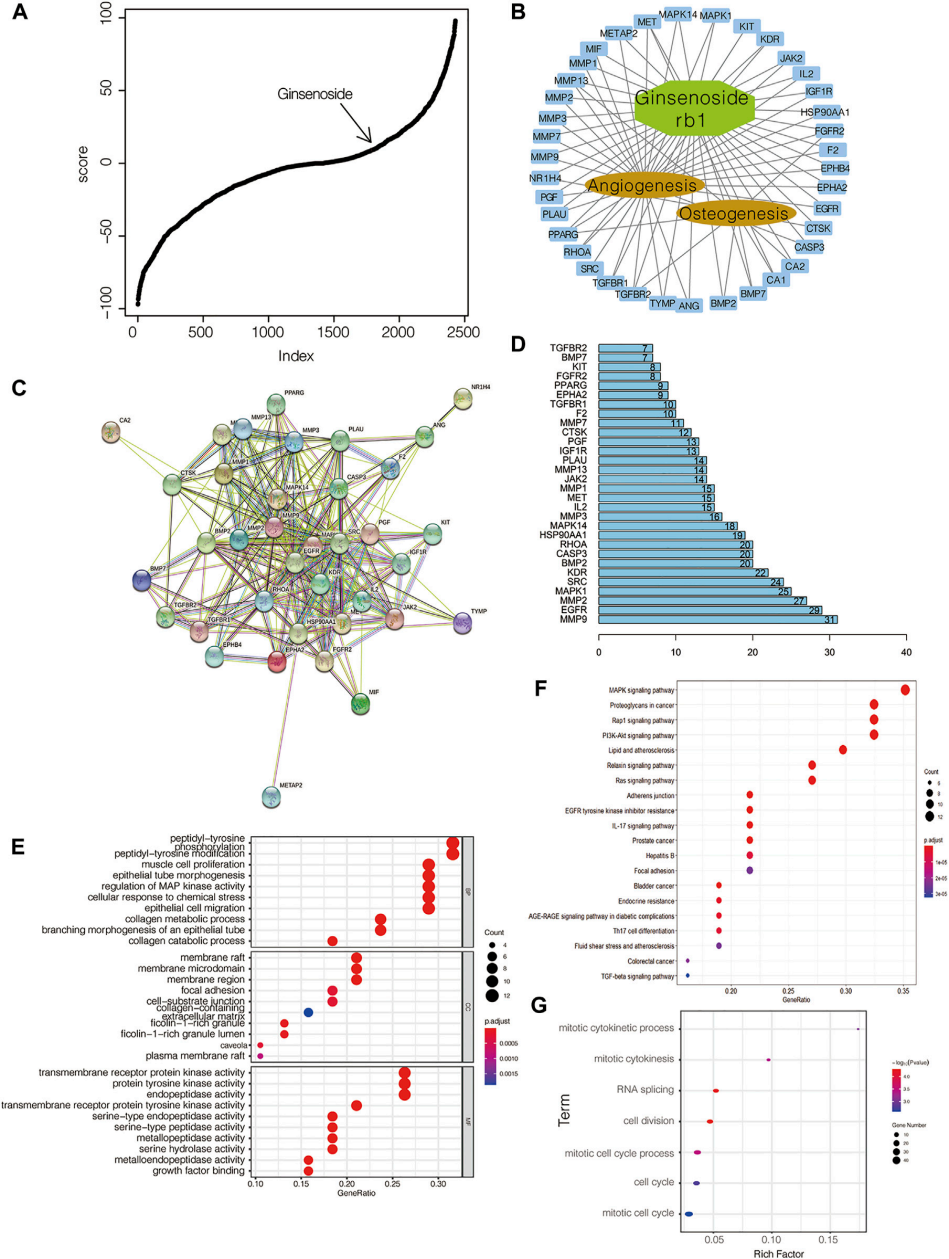
Identification of ginsenoside Rb1 as an osteogenic compound. **(A)** Ginsenoside Rb1 is involved in CMap-based molecular screening results from the icariin-induced gene expression signature; **(B)** network between ginsenoside Rb1-targeted genes and those related to osteogenesis and angiogenesis; **(C)** PPI between ginsenoside Rb1-targeted genes and those related to osteogenesis and angiogenesis; **(D)** hub genes in C; numbers on the bar and the edge number of the corresponding gene in C; **(E)** GO-based and **(F)** KEGG-based gene set enrichment analyses of genes in B; **(G)** GO-based gene set enrichment analysis of differentially expressed genes in BMSCs administered with ginsenoside Rb1 at 20 μM after 24 h treatment.

Since hydroxycholesterol, screened from the icariin-induced osteogenic gene expression model, has a potent effect on osteogenesis at an optimal concentration of 2 μM, we adopted this approach to find the effective osteogenic molecule to investigate if there were other natural compounds similar to icariin. On the basis of the aforementioned evidence, ginsenoside Rb1 was identified to have a similar gene expression profile to that of the icariin-induced osteogenic model (Figure 3A). By means of molecular docking, we obtained a series of ginsenoside Rb1-targeting proteins. By intersecting with proteins related to osteogenesis and angiogenesis, it was found that the effect of ginsenoside Rb1 may be partial to angiogenesis (Figure 3B). By PPI analysis and hub gene analysis, we identified BMP2, MMP9, and EGFR as the core proteins of the ginsenoside Rb1 target (Figures 3C,D). The result of functional gene enrichment analysis showed that ginsenoside Rb1-targeting proteins were mainly related to endothelial cell proliferation, cell adhesion, cell junction, MAPK signaling pathway, EGFR signaling pathway, tube morphogenesis, PI3K-AKT signaling pathway, etc. (Figures 3E,F). Indeed, with the gene expression profile of ginsenoside Rb1 being administered into BMSCs for 24 h at 20 μM, it was found that the over-represented GO terms were mainly involved in the cell cycle (Figure 3G), which is consistent with the predicted function of ginsenoside Rb1 mentioned earlier.

### Ginsenoside Rb1 enhanced osteogenic differentiation of BMSCs

**FIGURE 4:**
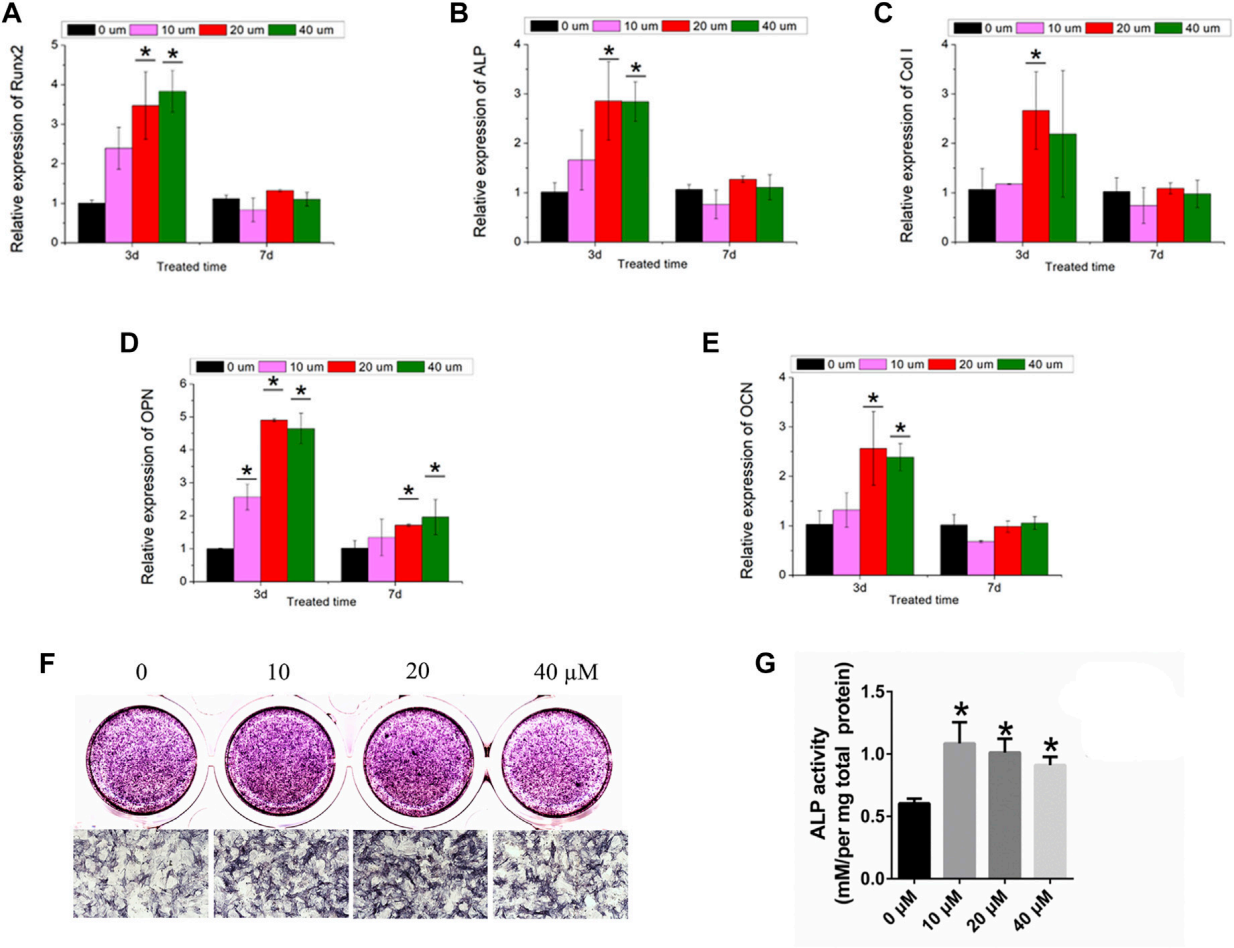
Osteogenic effect of ginsenoside Rb1. **(A–E)** Relative expressions of Runx2, ALP, Col-1, OPN, and OCN in BMSCs by different concentrations of ginsenoside Rb1 at days 3 and 7; **(F–G)** ALP staining and ALP quantitative activity assay of BMSCs administered with ginsenoside Rb1 (*, *p* < 0.05).

In the present study, the mRNAs of Runx2, ALP, Col-1, OPN, and OCN in BMSCs were detected by the treatment of ginsenoside Rb1 at the concentrations of 10, 20, and 40 µM (Figures 4A–E). It showed that the mRNA expressions of Runx2, ALP, Col-1, OPN, and OCN of BMSCs were enhanced by ginsenoside Rb1 after 3 days of treatment, and this increasing tendency slowed down as the treatment time extended (Figures 4A–E).

Moreover, ALP staining demonstrated that ginsenoside Rb1 significantly increased the ALP activity at 4 days (Figure 4F). Meanwhile, the ALP quantitative activity assay showed that ginsenoside Rb1 at 10, 20, and 40 μM concentrations could raise the ALP activity compared with the control group at day 4 in a dose-dependent manner (Figure 4G).

Taken together, the results proved that ginsenoside Rb1 was of great potential in promoting osteogenic differentiation of BMSCs.

### Ginsenoside Rb1 accelerated bone regeneration

**FIGURE 5:**
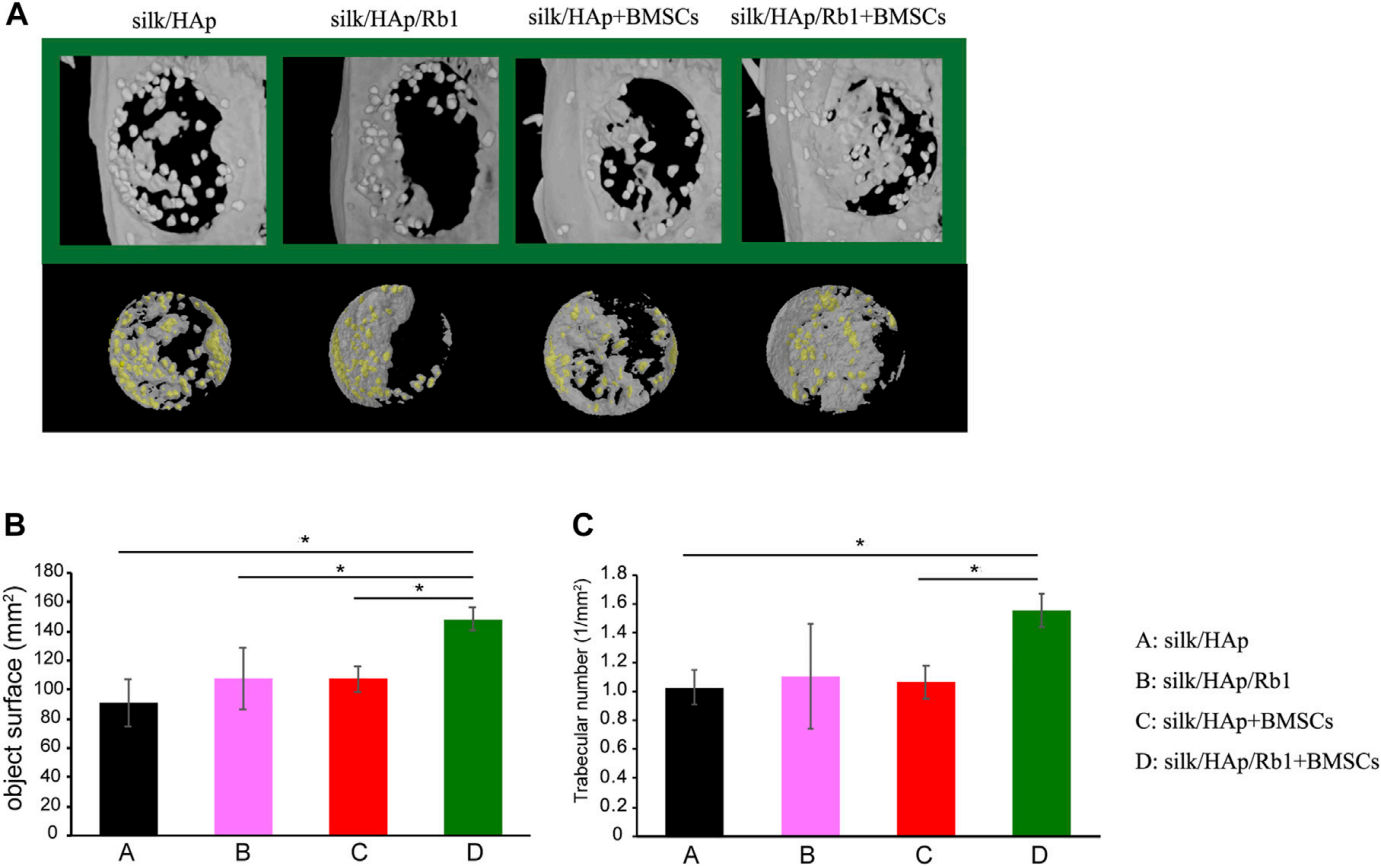
Micro-CT evaluation and morphometric analysis of calvarial defect repair. **(A)** Representative 3D superficial **(B)** object surface and **(C)** the trabecular number of calvarial defects were taken at 8 weeks after implantation, while the yellow color indicated the HAp granules (*, *p* < 0.05).

To investigate the osteogenic effect of ginsenoside Rb1 *in vivo*, a tissue-engineered silk fibroin hydrogel gel containing ginsenoside Rb1-loaded HAp granules was applied to the calvarial defect of rats to test its potential for bone regeneration. The micro-CT results showed that much more new bone formation was observed in the silk/HAp/Rb1+BMSCs group at 8 weeks after implantation (Figure 5A). The morphometrical analysis showed that a significantly greater object surface and trabecular number were detected in the silk/HAp/Rb1+BMSC group, which was higher than the other three groups, although there was no significant difference between the silk/HAp/Rb1 group and the silk/HAp/Rb1+BMSC group (Figures 5B,C).

## Discussion

Natural compounds are the treasure house of pharmacological research. They have attracted much attention because of their wide sources, low toxicity, and high utility (Newman and Cragg, 2020). A variety of natural compounds, including icariin, have been found to have osteogenic effects (Awale et al., 2017; Shi et al., 2022).

With the development of bioinformatic technology and through molecular docking and screening of the gene expression signature induced by a kind of pharmacological action, it has been gradually used to find other small molecules with similar actions in pharmacological research. Based on the icariin-induced gene expression profile in BMSCs, which has been confirmed to have a potent osteogenic effect, we conducted molecular screening to find more natural compounds with a potential osteogenic effect.

The approach used in this study has the following advantages: 1) reliability owing to the use of data from a known modality with a determined pharmacological action; 2) with the help of molecule docking and screening, the potential target proteins mediated by candidate compounds could be predicted precisely; and 3) the ability to identify the affected biological processes by functional gene enrichment analysis.

In order to verify the effectiveness of this method, we first performed the *in vitro* assay of hydroxycholesterol, which has one of the most similar gene expression profiles induced by icariin, and found that it has a potent osteogenic effect. At 7 days, the expression of genes related to osteogenic differentiation in BMSCs increased, and ALP staining confirmed its osteogenic effect (Figure 2). Hydroxycholesterol has been found to induce osteogenesis in human adipose-derived stem cells (Yalom et al., 2014) and mesenchymal stem cells (Bakshi et al., 2019). This osteoinductive effect was mediated by the hedgehog signaling pathway (Bakshi et al., 2019). The direct target of hydroxycholesterol, which was an agonist of the liver X receptor, may not be the same as icariin, but the changes in the gene expression profile are very similar to those induced by icariin and have osteogenic signatures.

Owing to the *in vitro* result of hydroxycholesterol, we verified that this approach of predicting related drugs through an osteogenic gene expression model is effective. Thus, among the compounds with similar gene expression signatures of icariin administration, we identified another natural compound, ginsenoside Rb1. Ginsenoside Rb1, the effective ingredient of ginseng, was found to have multiple bioactive features, such as antitumor, immunoregulation, antioxidative, and neuroprotective effects (Zhou et al., 2019; Ahmed et al., 2016; Xie et al., 2021). We used pharmacological network analysis and molecular docking to predict the pharmacological effect of ginsenoside Rb1, and found that it is concentrated on osteogenesis and angiogenesis and partial to angiogenesis. Through subsequent experiments *in vivo* and *in vitro*, we also verified this conjecture. Although ginsenoside Rb1 has a certain osteoinductive effect, it is weaker than icariin. Otherwise, we found that the angioinductive effect of ginsenoside Rb1 is also prominent, as illustrated in this study and our recent work (Wu et al., 2022). Previous studies have demonstrated the role of ginsenoside Rb1 in bone homeostasis, which includes preventing bone/cartilage destruction and therapeutic properties for osteoporosis (Hossain et al., 2022; Zhu et al., 2016). However, the detailed pathway related to the angioinductive effect mediated by ginsenoside Rb1 remains unclear. From the results of our bioinformatic analysis, the candidates seemed to be EGFR, MAPK, and/or PI3K-AKT signaling pathways (Figures 3D,E).

## Conclusion

In the present study, we used a computer simulation method to widely screen the osteogenic gene expression profiles and obtained a series of potential small molecular compounds with osteogenic effects. Their osteogenic effects were confirmed by experiments *in vivo* and *in vitro*. We provided an important reference for the subsequent research and development of medications for bone repair and regeneration.

## Data Availability

The accession number for the RNA-sequencing datasets of Ginsenoside Rb1-treated BMSCs presented in this article is GSE207667 (Gene Expression Omnibus). The accession number for microarray datasets of Icariin-treated BMSCs presented in this article is GSE208583 (Gene Expression Omnibus).
